# Respi-Radar: a tool to monitor respiratory infections, Belgium, winter season 2023/24

**DOI:** 10.2807/1560-7917.ES.2025.30.35.2400756

**Published:** 2025-09-04

**Authors:** Géraldine De Muylder, Simon Couvreur, Giulietta Stefani, Claire Brugerolles, Yinthe Dockx, Milena Callies, Raphaël Janssens, Laurane De Mot, Nathalie Bossuyt, Jorgen Stassijns

**Affiliations:** 1 Epidemiology of Infectious Diseases, Sciensano, Brussels, Belgium; 2 Healthcare-associated infections and antimicrobial resistance, Sciensano, Brussels, Belgium; 3 Crisis Preparedness and Response, Sciensano, Brussels, Belgium

**Keywords:** COVID-19, respiratory infections, management tool, policies

## Abstract

Following the experience gained during the COVID-19 pandemic, the Belgian Risk Assessment Group (RAG) developed the Respi-Radar in the summer of 2023 to assess the epidemiological situation of respiratory infections and inform public health preparedness and response in Belgium. The Respi-Radar consists of four risk levels (green, yellow, orange and red), which indicate the extent of viral circulation and/or pressure on the healthcare system. Based on these risk levels, authorities can apply adequate measures depending on the epidemiological trends. The Respi-Radar uses six indicators from the influenza-like illness and severe acute respiratory infection sentinel surveillances in nursing homes, primary and secondary healthcare and wastewater surveillance. Additional information such as data from the national reference laboratory is also used to assess the epidemiological situation. Using the Respi-Radar tool, the RAG regularly evaluated the epidemiological situation of respiratory infections between September 2023 and March 2024. The Respi-Radar tool was useful for following epidemiological trends and effectively communicate the epidemiological situation of respiratory infections. Linking specific measures to each risk level was less straightforward. The experience gained using the Respi-Radar tool was key in determining an appropriate approach to assess and manage the epidemiological situation for future respiratory seasons.

## Background

In Belgium, as in other countries, the COVID-19 pandemic led to the enhancement of existing surveillance systems and the development of novel structures. This resulted in unprecedented amounts and types of data available. Management tools were created to evaluate the COVID-19 epidemiological situation on a regular basis and in a structured way. These management tools incorporated key indicators and thresholds to define risk levels, which would ultimately offer guidance to policymakers [1]. In addition, these management tools aimed to link each risk level with specific measures to mitigate the spread of COVID-19.

The management tools were successively used for monitoring COVID-19 in Belgium between September 2020 and August 2023 as previously described [1]. They were developed by the Risk Assessment Group (RAG), an independent scientific expert group coordinated by the Belgian Institute of Health, Sciensano, and composed of representatives of the federal and regional public health authorities as well as professionals selected based on their expertise (epidemiologists, clinicians, microbiologists, hygienists, biostatisticians) [2]. The latest COVID-19 management tool, from December 2021, distinguished three risk levels: (i) epidemiological situation under control (Level 1), (ii) increasing viral circulation potentially leading to pressure on the healthcare system (Level 2), and (iii) high viral circulation with possible healthcare system overload (Level 3). The levels were based on several indicators, essentially reflecting COVID-19-related pressure on the healthcare system (number of hospital admissions due to COVID-19, COVID-19-related intensive care unit (ICU) occupancy and number of general practitioner (GP) consultations for suspicion of COVID-19), as well as other indicators such as COVID-19 positivity rate for symptomatic patients, reproductive number (Rt) and 14-day incidence of cases per 100,000 population. Although translating risk levels into specific public health measures was challenging, the tool allowed for a clear and simple communication to authorities, the healthcare sector and the public.

Due to the cocirculation of severe acute respiratory syndrome coronavirus 2 (SARS-CoV-2) and other respiratory pathogens, it became necessary to develop a tool that would take into account not only SARS-CoV-2 but also other respiratory pathogens, and which would include an additional level reflecting a non-epidemic situation. The ‘Respi-Radar’ tool was introduced in the summer of 2023 with the aim of assessing the epidemiological situation of the main respiratory infections and informing public health preparedness and response. This system was primarily intended for Belgian authorities and the healthcare sector, but it also assisted with communication to the public.

This perspective first describes the Respi-Radar tool, its implementation and how it was used during the 2023/24 respiratory infection season. It then presents a brief evaluation of the tool and discusses its advantages and limitations.

## Description of the Respi-Radar tool

### Indicators used

The Respi-Radar is based on five pathogen-agnostic indicators from sentinel surveillance systems of respiratory infections, as well as one SARS-CoV-2-specific indicator. These indicators were chosen, following discussion within the RAG expert group, to cover the circulation of respiratory pathogens (weekly incidence of GP consultations for influenza-like illness (ILI), weekly incidence of GP consultations for other acute respiratory infections (ARI), concentration of SARS-CoV-2 in wastewater), the severity of diseases (weekly incidence of ILI cases in nursing homes, weekly incidence of hospitalisation for severe acute respiratory infections (SARI), weekly incidence of SARI hospitalisation with severe complications) and their impact on the healthcare system. The indicators are listed in Table 1. The composition of the RAG expert group for the 2023/24 season is described in Supplementary Table S1.

**Table 1:** Indicators used in the Respi-Radar, Belgium, 2023–2024 (n = 6 indicators)

Indicators	Data source	Reporting time lag	Reference
Weekly incidence of GP consultations for ILI per 100,000 inhabitants	Sentinel network of GPs (routinely collected)	1 week	[8]
Weekly incidence of GP consultations for other ARI per 100,000 inhabitants	Sentinel network of GPs (routinely collected)	1 week	[8]
Weekly incidence of ILI cases in nursing homes per 1,000 nursing home residents	Sentinel network of nursing homes (routinely collected)	1 week	[9]
Weekly incidence of hospitalisation for SARI per 100,000 inhabitants	Sentinel network of hospitals (routinely collected)	2 weeks	[8]
Weekly incidence of SARI hospitalisation with severe complications (invasive ventilation, ECMO, admission to ICU, ARDS, death) per 100,000 inhabitants	Sentinel network of hospitals (routinely collected)	Length of hospitalisation plus 4 weeks	[8]
Number of wastewater treatment plants with high concentrations of SARS-CoV-2	Wastewater surveillance (routinely collected)	0	[10]

ARDS: acute respiratory distress syndrome; ARI: acute respiratory infection; ECMO: extracorporeal membrane oxygenation; GP: general practitioner; ICU: intensive care unit; ILI: influenza-like illness; SARI: severe acute respiratory infection; SARS-CoV-2: severe acute respiratory syndrome coronavirus 2.

### Definition of indicator thresholds

Different thresholds were defined for each indicator. For the incidence of GP consultations for ILI, the incidence of GP consultations for ARI and the incidence of hospital admissions for SARI, the thresholds were defined by the Moving Epidemic Method (MEM), with support from the European Centre for Disease Control and Prevention (ECDC) for ILI. The MEM is an exceedance-based system which uses historical data from past epidemic waves to identify potential upcoming epidemics and assess their intensity [3]. For the incidence of ILI cases in nursing homes and the number of wastewater treatment plants with a high concentration of SARS-CoV-2, historical data were insufficient to use the MEM. Thresholds were therefore set by expert consensus. All thresholds are summarised in Table 2.

**Table 2:** Risk levels in the Respi-Radar and thresholds defined for each indicator, Belgium, 2023–2024 (n = 4 levels)

Risk level	GP consultations for ILI^a^	GP consultations for ARI^a^	ILI in nursing homes^b^	Hospitalisations for SARI^a^	Complications linked to SARI^c^	SARS-CoV-2 wastewater^d^
Green	< 128	< 1,208	< 7	< 4.4	< 0.68	< 5
Yellow	128–507	1,208–1,293	7–13	4.4–9.8	0.68–1.4	5–10 stations
Orange	508–783	1,294–1,984	14–20	9.9–33.7	1.41–3.03	11–15 stations
Red	> 783	> 1,984	> 20	> 33.7	> 3.03	> 15 stations

ARI: acute respiratory infection; ILI influenza-like illness; SARI: severe acute respiratory infection; SARS-CoV-2: severe acute respiratory syndrome coronavirus 2.

^a^ Weekly incidence per 100,000 inhabitants.

^b^ Weekly incidence per 1,000 nursing home residents.

^c^ Weekly incidence per 100,000 inhabitants. A complication is defined as death, acute respiratory distress syndrome, admission to intensive care unit, extracorporeal membrane oxygenation or invasive ventilation.

^d^ Number of treatment plants with high concentrations of SARS-CoV-2 as defined in [10].

### Definition of Respi-Radar risk levels

The Respi-Radar was divided into four risk levels (Table 2). The green level was the baseline and reflected a non-epidemic situation. The yellow level represented a situation where the epidemic threshold was passed for some indicators, pathogens were circulating, but the impact on the healthcare system remained limited. The orange level was characterised by a moderate pathogen circulation, which exerted moderate pressure on the healthcare system (for instance increased consultations at GP practices, increased hospitalisations). The red level reflected a situation where pathogen circulation was high, with an expectation of healthcare system overload. The thresholds set for each indicator determined the Respi-Radar levels.

### The Respi-Radar workflow

Data from the surveillance of respiratory infections (Table 1) were available once a week and presented in the weekly bulletin of acute respiratory infections [4].

Every week, experts from the RAG analysed the indicators included in the Respi-Radar and estimated an overall risk level which characterised the epidemiological situation for the week under review. The assessment was based on the Respi-Radar indicators, but other relevant information could also be included when needed, for instance the European epidemiological situation (through the European Respiratory Virus Surveillance Summary (ERVISS) [5]), genomic surveillance data from the national reference laboratory or information provided by the RAG experts. The decision of the overall risk level was thus based on both quantitative and qualitative information and taken by consensus by the RAG experts. In addition to estimating risk level, the RAG could propose appropriate actions, such as emphasising the importance of vaccination, basic protective measures or reinforcing communication with relevant groups.

The risk level and actions proposed by the RAG were then presented weekly to the Risk Management Group (RMG) composed of representatives from health authorities and coordinated by the Ministry of Health. The RMG validated the analysis and decided on measures to be applied [6]. Specific sets of measures adapted to each Respi-Radar risk level were developed by other advisory bodies, such as the Strategic Scientific Committee [7].

## Use of the Respi-Radar tool for respiratory infections in 2023/24

**Figure 1:**
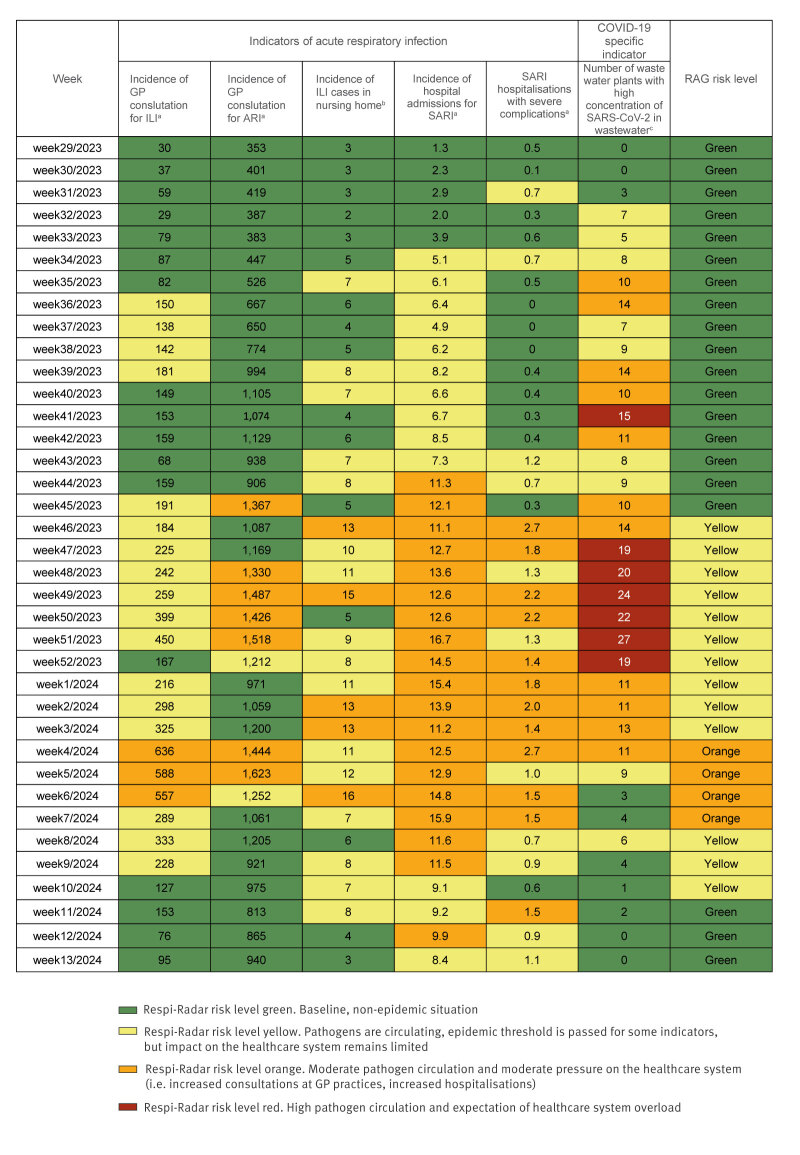
Respi-Radar evaluation, Belgium, July 2023–March 2024 (n = 37 weeks)

ARI: acute respiratory infection; COVID-19: coronavirus disease 2019; GP: general practitioner; ILI influenza-like illness; RAG: Risk Assessment Group; SARI: severe acute respiratory infection; SARS-CoV-2: severe acute respiratory syndrome coronavirus 2.

^a^ Weekly incidence per 100,000 inhabitants.

^b^ Weekly incidence per 1,000 nursing home residents.

^c^ Number of wastewater treatment plants with high concentrations of SARS-CoV-2 as defined in [10].

The epidemiological situation for respiratory infections was assessed as being at baseline (Risk level green) between July and mid-November 2023 (weeks 29–45). From 23 November 2023 (week 46) to 21 January 2024 (week 3), the situation was assessed as being at Risk level yellow because several indicators showed increasing epidemiological trends. Nevertheless, the situation was under control with a limited impact on healthcare. This increase was due to a rise in respiratory syncytial virus (RSV) circulation in October 2023, followed by increased SARS-CoV-2 circulation in December. From 22 January 2024 (week 4) to 18 February 2024 (week 7), the epidemiological situation for respiratory infections was at Risk level orange, mainly due to the increased circulation of influenza and the subsequent pressure on healthcare. The epidemiological situation was assessed as being at Risk level yellow again on 19 February 2024 (week 8) because the impact on the healthcare system was limited, despite viral circulation above the epidemic threshold. From 11 March 2024 (week 11) and until the end of the respiratory infection season, the epidemiological situation for respiratory infections remained at baseline (Figure 1).

In accordance with the estimated risk level, the RAG proposed several actions over time. In September 2023, the risk level was green, but in light of the upcoming respiratory season, the RAG recommended that the existing guidelines for managing cases of COVID-19 and respiratory infections in general would be reiterated. In October 2023, still at Risk level green, the RAG stressed the importance of maintaining basic protective measures (washing hands, staying at home or wearing a mask in case of symptoms). The RAG also emphasised the importance of vaccination against respiratory pathogens (influenza, SARS-CoV-2, RSV and pneumococcus) for vulnerable populations. In November 2023, when the risk level was increased to yellow, the RAG referred to the recommendations formulated by other advisory bodies for the general population in Belgium: stay at home in case of symptoms; ventilate indoor spaces; wear a mask in case of symptoms; and vaccinate at-risk populations against respiratory pathogens. Recommendations for the healthcare sector were proposed by the RMG. In January 2024, when the risk level was raised to orange, no additional measures were set, but the RMG reinforced communication to the public and the healthcare sector to reiterate the measures described above and stress the importance of ventilation and protection of vulnerable people.

## Evaluation of the Respi-Radar

In spring 2024, the RAG conducted an evaluation of the Respi-Radar, which included an analysis of the relevance of the chosen indicators, timely availability of data, adequacy of the thresholds and actions taken. The evaluation was based on two components: (i) an analysis of the dynamics of each indicator monitored within the Respi-Radar during the 2023/24 season and their alignment with the global level defined each week, and (ii) a survey carried out among the experts participating in the RAG.

### Analysis of the Respi-Radar indicators

**Figure 2:**
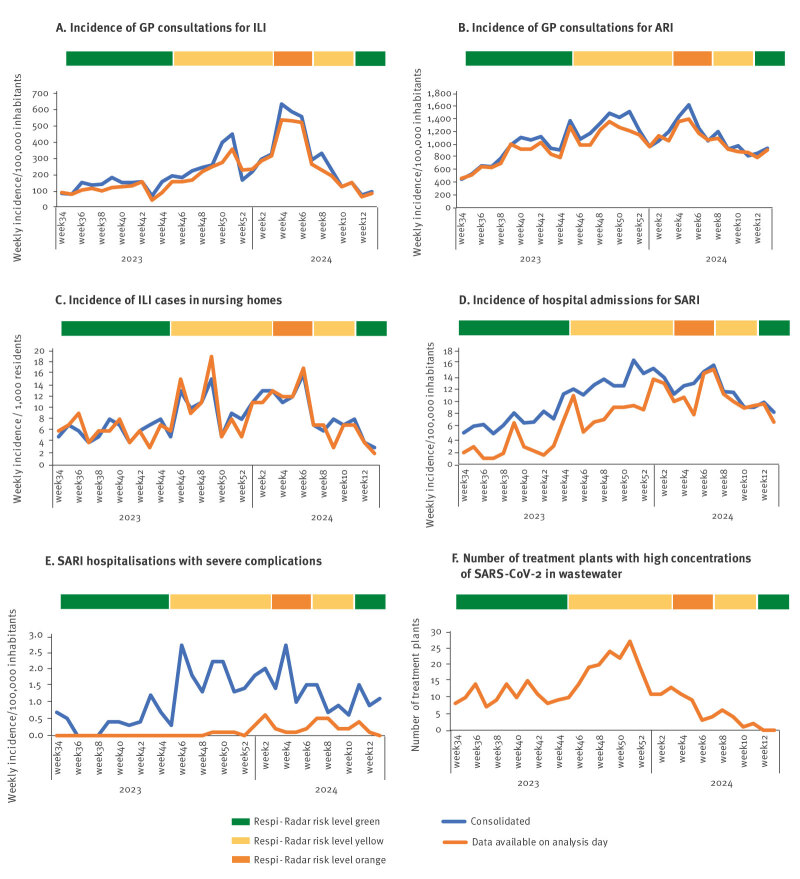
Analysis of the indicators used in the Respi-Radar, Belgium, week 34 2023–week 13 2024

ARI: acute respiratory infection; GP: general practitioner; ILI influenza-like illness; SARI: severe acute respiratory infection; SARS-CoV-2: severe acute respiratory syndrome coronavirus 2.

The completeness of the data available for the Respi-Radar evaluation was assessed for each indicator by comparing the data available at the time of the evaluation and the data when consolidated (Figure 2).

Panels A, B, D and E are weekly incidences per 100,000 population. Panel C is weekly incidence per 1,000 nursing home residents. Panel F is as defined in [10].

Data available at the time of the Respi-Radar analysis (the orange line) were compared with the same data when consolidated (blue line) from week 34 2023 to week 13 2024. Coloured boxes (green, yellow or orange) indicate the overall risk level defined weekly through the Respi-Radar.

Data from the sentinel networks of GPs and nursing homes were slightly underestimated or overestimated on the day of the Respi-Radar evaluation, with stronger underestimation at epidemiological peaks (Figures 2A, 2B and 2C). However, the epidemiological trends were correctly captured on the day of the evaluation. The incidence of hospital admissions for SARI (from the sentinel network of hospitals) was underestimated between November and December 2023 due to several changes in the surveillance system (expansion of the sentinel network, change in the data collection system). From January 2024, surveillance from hospitals greatly improved and reflected the epidemiological trends observed in the data from the sentinel network of GPs (Figure 2D). On the other hand, the incidence of SARI hospitalisations with severe complications, collected from the sentinel network of hospitals, was never available at the time of the Respi-Radar evaluation. Since this indicator is collected at patient discharge, it typically requires 4­–5 weeks to be consolidated (Figure 2E). The number of wastewater treatment plants positive for SARS-CoV-2 was always available at the time of the Respi-Radar evaluation (Figure 2F).

To assess the relevance of each indicator (i.e. their importance in determining the overall risk level), the dynamics of each indicator were examined in parallel to the Respi-Radar overall risk level over time (Figure 2).

For four indicators (incidence of consultations at GP practices for ILI symptoms; incidence of consultations at GP practices for ARI; incidence of hospital admissions for SARI; and incidence of ILI in nursing homes), the dynamics taken individually matched the Respi-Radar overall evaluation in that increasing epidemiological trends were observed when the risk level was changed from green to yellow and then from yellow to orange. Conversely, the trend decreased when the Respi-Radar risk level changed from orange to yellow and then from yellow to green. The incidence of complications after hospitalisation for SARI was not available at the time of the Respi-Radar analysis, but consolidated data showed a good match with the overall Respi-Radar evaluation.

The indicator on viral concentrations in wastewater did not match the Respi-Radar evaluation, as, during the period considered here (July 2023–March 2024), this indicator remained pathogen-specific, with only SARS-CoV-2 concentrations measured in wastewater while the Respi-Radar evaluated respiratory infections in general.

### Feedback from the experts of the Risk Assessment Group

A survey was sent to the RAG experts in April 2024 asking them for feedback on the selection of indicators within the Respi-Radar, the epidemiological thresholds defined for these indicators, the correspondence between the Respi-Radar level and the epidemiological situation and the usefulness of the Respi-Radar as a decision supporting tool. The Supplementary Table S2 shows the list of questions that were asked to the RAG experts.

Eighteen of the 26 RAG experts responded to the survey (Supplementary Figure S1 shows the participation of RAG experts to the survey, by domain of expertise). The indicators and thresholds chosen were generally considered adequate, with the exception of the incidence of severe complications after hospitalisation for SARI, for which the delay was considered too long to obtain reliable data. Several experts (11/18) suggested pathogen-specific indicators be included in the tool. The Respi-Radar was seen by the experts as a useful tool for monitoring trends in respiratory infections in a standardised manner and for clear communication of the epidemiological situation to the authorities, the healthcare sector and the general public. However, applying a predefined set of measures to each determined risk level was challenging, so the decision-supporting aspect of the tool was less recognised (Supplementary Figure S2 details the results of the survey).

## Advantages and limitations of the Respi-Radar tool

Belgium, as most European countries, publishes an extensive weekly epidemiological report for acute respiratory infections, which contains the results of syndromic surveillances as well as pathogen-specific data from a network of laboratories, GP practices, hospitals and wastewater.

The added value of the Respi-Radar tool, as compared with the executive summary of an epidemiological report, was that: (i) it provides a standardised procedure to evaluate the epidemiological situation, and (ii) by summarising the results from multiple surveillances/indicators in a single level, it offers a clear message to authorities, the healthcare sector and the general population.

The Respi-Radar tool also has some limitations. The first limitation is the use of thresholds based on historical data from each surveillance system. In some cases, these data may be incomplete, or system changes may create inconsistencies. Furthermore, thresholds that are effective during non-pandemic periods may not be as useful during pandemics. Even in syndromic surveillance, the occurrence of a new pathogen and disruptions to the seasonality of known pathogens can result in unusually high peaks, complicating the detection of changing risks. Therefore, thresholds should be interpreted with caution, requiring expert judgment. The second limitation is that the Respi-Radar provides a snapshot of the prevailing epidemiological situation, but it might be too late at that moment to apply efficient mitigation measures due to the delay in obtaining consolidated data as well as the lagged effect of these measures.

A third limitation of the Respi-Radar is that the level of virus circulation measured in wastewater during the 2023/24 season was specifically for SARS-CoV-2. This was due to the fact that other respiratory viruses were not monitored in wastewater during the period considered here (winter 2023/24). This led to discrepancies between the overall Respi-Radar level and the indicator on SARS-CoV-2 circulation in wastewater. An objective for the next respiratory seasons is to obtain a global indicator for respiratory virus circulation in wastewater that encompasses SARS-CoV-2, influenza A, influenza B, RSVA and RSVB.

Finally, a characteristic of the Respi-Radar is that it is based on a mix of quantitative data, qualitative information from experts and other relevant information including pathogen-specific data when needed. The Respi-Radar tool is thus not exclusively a quantitative system following a predetermined algorithm and a discussion with experts is crucial to determine the weekly overall risk level.

## Conclusion

The Respi-Radar was implemented in July 2023 and was used during the autumn-winter 2023/24 season in Belgium. This season was characterised by an influenza epidemic of moderate intensity and relatively long duration (11 weeks), a COVID-19 peak with limited impact in December 2023 and an RSV peak in October 2023 with an intensity comparable to the previous year. No measures were imposed by the authorities on the healthcare sector or the population, but recommendations were formulated.

The Respi-Radar was built from management tools developed during the COVID-19 pandemic to inform and guide policymakers. It aims to provide a standardised and integrated evaluation of the epidemiological situation of respiratory infections in Belgium, ensuring a system for early warning and facilitating communication to authorities, the healthcare sector and the general population. The Respi-Radar could be of significant interest to international public health organisations and other countries due to its alignment with ECDC recommendations on respiratory virus surveillance. Due to its flexibility, the method could be adopted by countries with differing healthcare infrastructures and surveillance capacities, aiding in building a more resilient international surveillance network.

In line with the conclusions drawn during the review of the management tools previously developed for COVID-19, challenges remain to link predefined sets of recommendations to the specific risk levels of the Respi-Radar tool.

The Respi-Radar will be used in the coming respiratory infection seasons in Belgium. Adaptations to improve the detection and monitoring of respiratory infections will include: (i) changes in the reporting scheme from hospitals, improving hospital data timeliness, and (ii) monitoring of other respiratory viruses (influenza, RSV) from wastewater surveillance. Adaptations to establish risk level-based sets of recommendations in a standardised way are also foreseen. These recommendations will be decided and implemented by the Belgian health authorities, based on the Respi-Radar (epidemiological situation) but also on other aspects such as availability of resources or acceptance by society. Measures should be implemented in a timely manner to help mitigate and control pressure on the healthcare system.

## Data Availability

Not applicable.

## Supplementary Data

333000 Supplementary Material
