# Supplementary material for: Respi-Radar: a tool to monitor respiratory infections, Belgium, winter season 2023/24
Source: Euro Surveill. 2025 Sep 4;30(35):2400756. doi: 10.2807/1560-7917.ES.2025.30.35.2400756 (PMC12413606; doi:10.2807/1560-7917.ES.2025.30.35.2400756)
Supplement: Supplementary Material [file 24-00756_DE_MUYLDER_Supplement.pdf]

Supplementary material

This supplementary material is hosted by *Eurosurveillance* as supporting information alongside the article 'Respi-Radar: a tool to monitor respiratory infections, Belgium, winter season 2023/24', on behalf of the authors, who remain responsible for the accuracy and appropriateness of the content. The same standards for ethics, copyright, attributions and permissions as for the article apply. Supplements are not edited by *Eurosurveillance* and the journal is not responsible for the maintenance of any links or email addresses provided therein.

Supplementary Table S1: Profile of the experts of the RAG, Belgium, 2023-2024

**Profile**	**Average presence per meeting**	**Percentage average presence**
Public health authorities (federal)	3	13 %
Public health authorities (federated entities)	4	18 %
Primary care	3	13 %
Hospital	4	17 %
University-Academic	3	13 %
Public health epidemiology (Sciensano)	6	26 %
total	23	100 %

RAG: Risk Assessment Group

Supplementary Table S2: List of questions asked to the experts of the RAG, Belgium, April 2024

	**Question**	**Answer options**
**Q1**	**Do you think that the indicators used in the Respi-Radar are relevant for the evaluation of the epidemiological situation of respiratory infections?**	Scale 1 (very irrelevant) to 5 (very relevant)
	Incidence of consultations at GP practices for ILI symptoms (weekly incidence/100 000 inhabitants)	
	Incidence of consultations at GP practices for ARI (weekly incidence/100 000 inhabitants)	
	ILI in nursing homes (weekly incidence/1000 nursing home residents)	
	Incidence of hospitalisations for SARI (weekly incidence/100 000 inhabitants)	
	Severe complications after hospitalisation for SARI (weekly incidence/100 000 inhabitants)	
	SARS-CoV-2 concentrations in wastewater (number of treatment plants positive for indicator "high circulation")	
**Q2**	**Is the current set of indicators within the Respi-Radar sufficient to capture the epidemiological situation of respiratory infections?**	Yes/No + comment box
**Q3**	**Do you think the Respi-Radar table should include one or more pathogen-specific indicators?**	Yes/No + comment box
**Q4**	**Do you think the thresholds are set adequately for each of the Respi-Radar indicators?**	Yes/No + comment box
**Q5**	**Are the Respi-Radar indicators sufficiently timely to evaluate the epidemiological situation?**	Yes/No + comment box

**Q6**	**Is it sufficiently clear what the completeness of the data is when the evaluation is done?**	Yes/No + comment box
**Q7**	**Do you think it would be useful to have a region-specific Respi-Radar tool in addition to the national Respi-Radar?**	Yes/No + comment box
**Q8**	**The decision process is based on quantitative (indicators) and qualitative information (experts feedback). Do you think the relative weight given to each is appropriate?**	Scale 1 (Significantly too much emphasis on quantitative information) to 5 (Significantly too much emphasis on qualitative information)
**Q9**	**Please indicate whether you agree or disagree with following statements**	Scale 1 (fully agree) to 5 (fully disagree)
	The Respi-Radar level accurately reflects the real situation in the community	
	The Respi-Radar level accurately reflects the real situation at the level of general practitioners	
	The Respi-Radar level accurately reflects the real situation in hospitals	
	The Respi-Radar level accurately reflects the real situation in nursing homes	
**Q10**	**Do you think it is useful to have a tool such as the Respi-Radar, to summarize the epidemiological situation in a "normal" influenza season?**	Scale 1 (very useful) to 4 (not useful at all)
**Q11**	**Do you think it is useful to have a tool such as the Respi-Radar, to summarize the epidemiological situation in an unexpected or unusual situation?**	Scale 1 (very useful) to 4 (not useful at all)
**Q12**	**Do you think it is useful to have a tool such as the Respi-Radar, to guide the decision process about control measures in a "normal" influenza season?**	Scale 1 (very useful) to 4 (not useful at all)
**Q13**	**Do you think it is useful to have a tool such as the Respi-Radar, to guide the decision process about control measures in an unexpected or unusual situation?**	Scale 1 (very useful) to 4 (not useful at all)

ARI: acute respiratory infection; GP: general practitioner; ILI influenza-like illness; RAG: Risk Assessment Group; SARI: severe acute respiratory infection; SARS-CoV-2: severe acute respiratory syndrome coronavirus 2

Supplementary Figure S1: Participation to the survey of RAG members by domain of expertise, Belgium, April 2024 (n=26 experts asked)

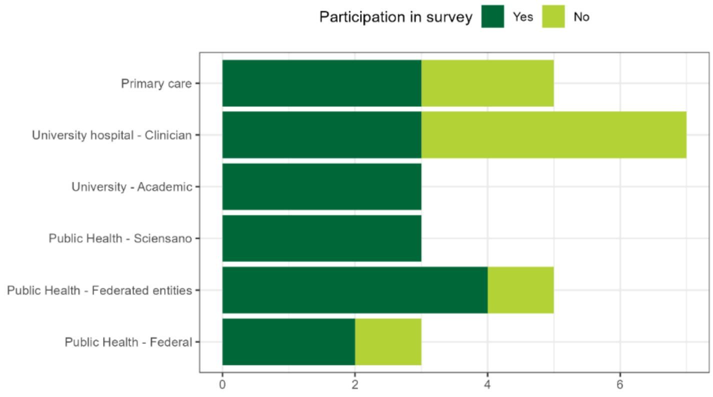

RAG: Risk Assessment Group

Supplementary Figure S2: Feedback from RAG experts, Belgium, April 2024 (n=5 panels)

A- relevance of the indicators used within the Respi-Radar; B- indicators and thresholds; C- accuracy of the Respi-Radar; D- decision process; E- usefulness of the Respi-Radar tool

A

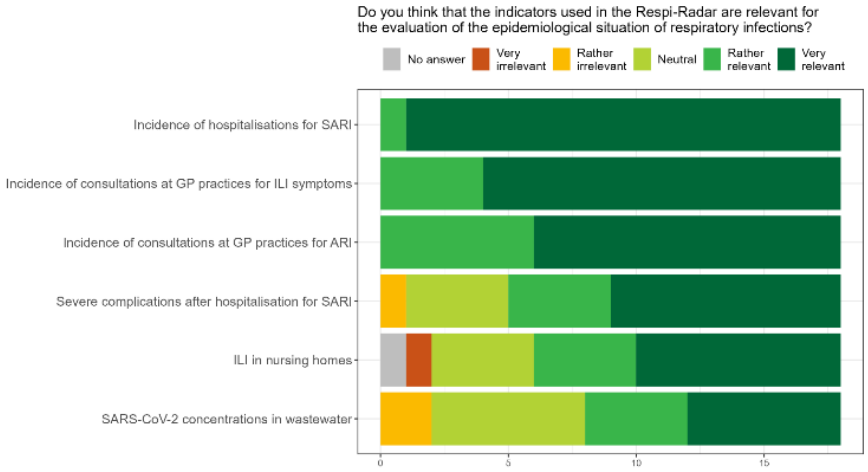

B

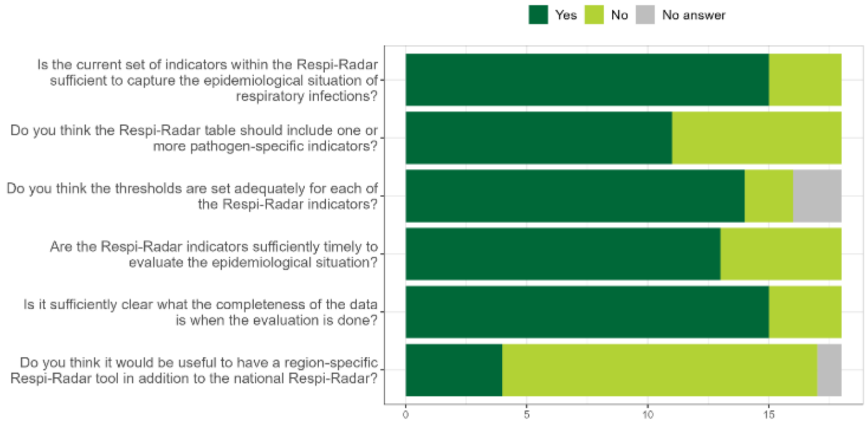

C

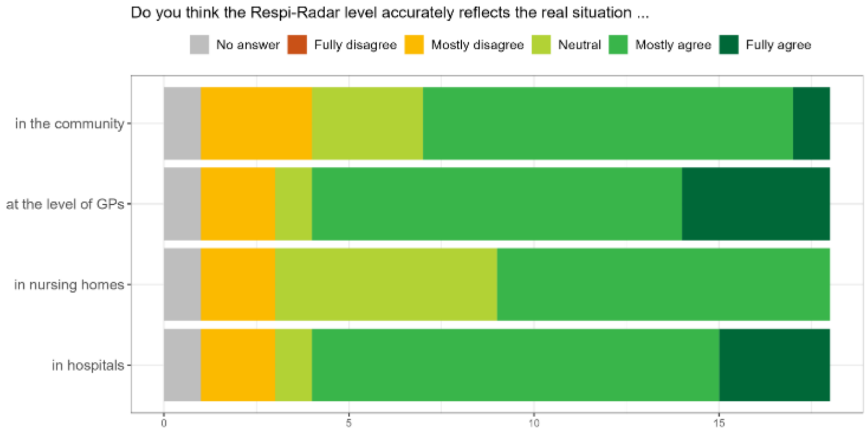

D

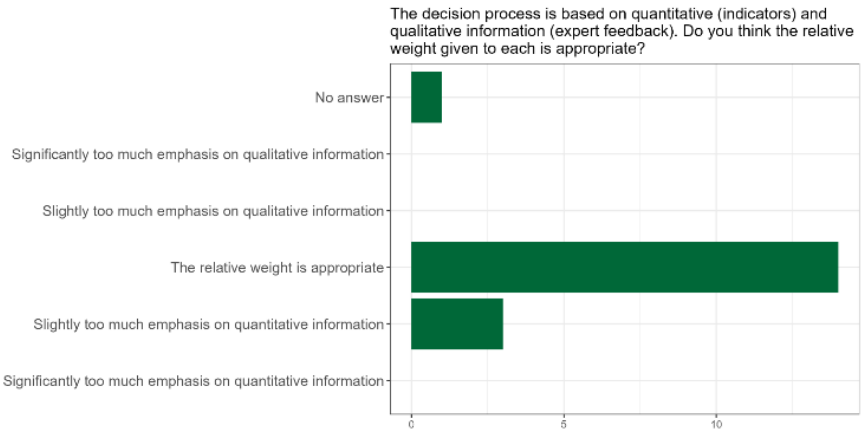

E

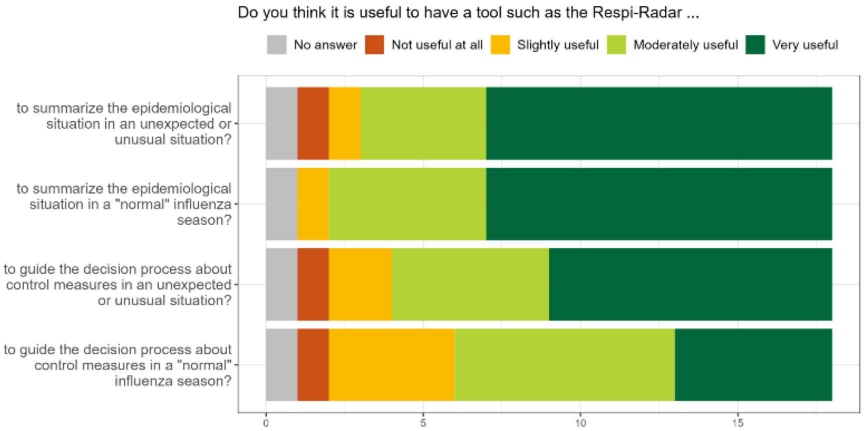

ARI: acute respiratory infection; GP: general practitioner; ILI influenza-like illness; RAG: Risk Assessment Group; SARI: severe acute respiratory infection; SARS-CoV-2: severe acute respiratory syndrome coronavirus 2
